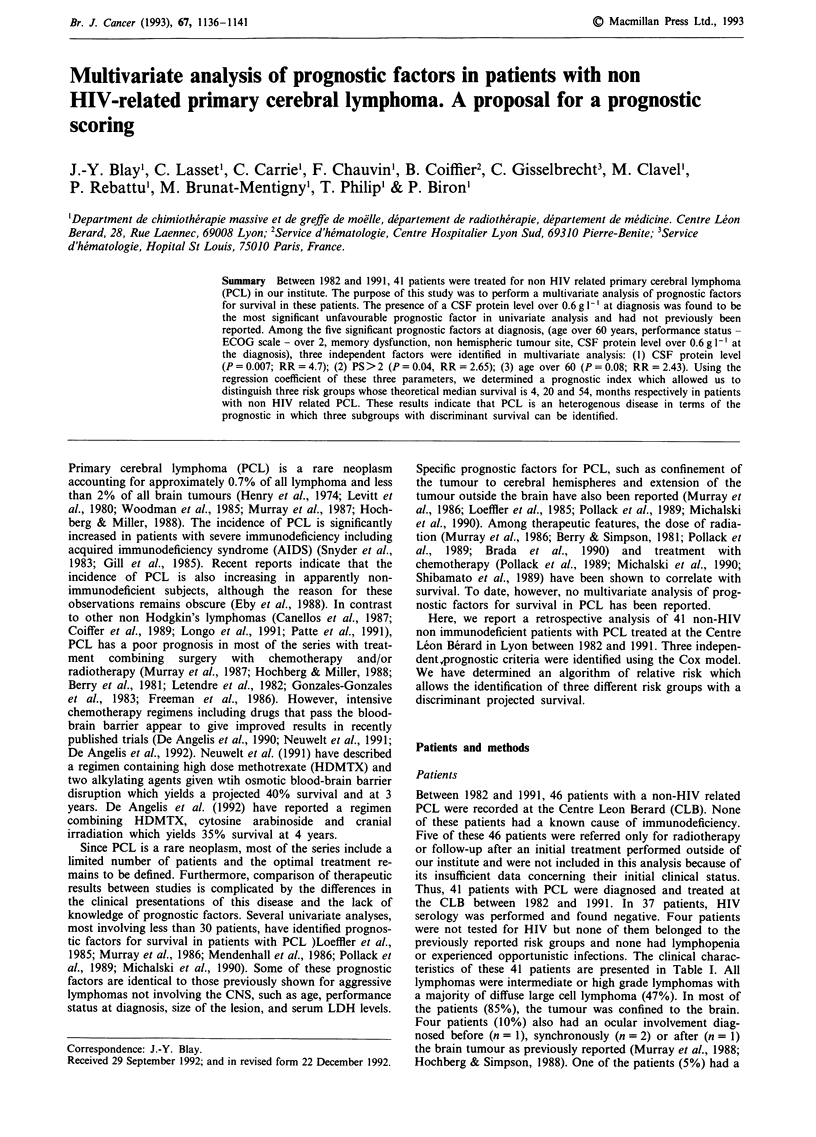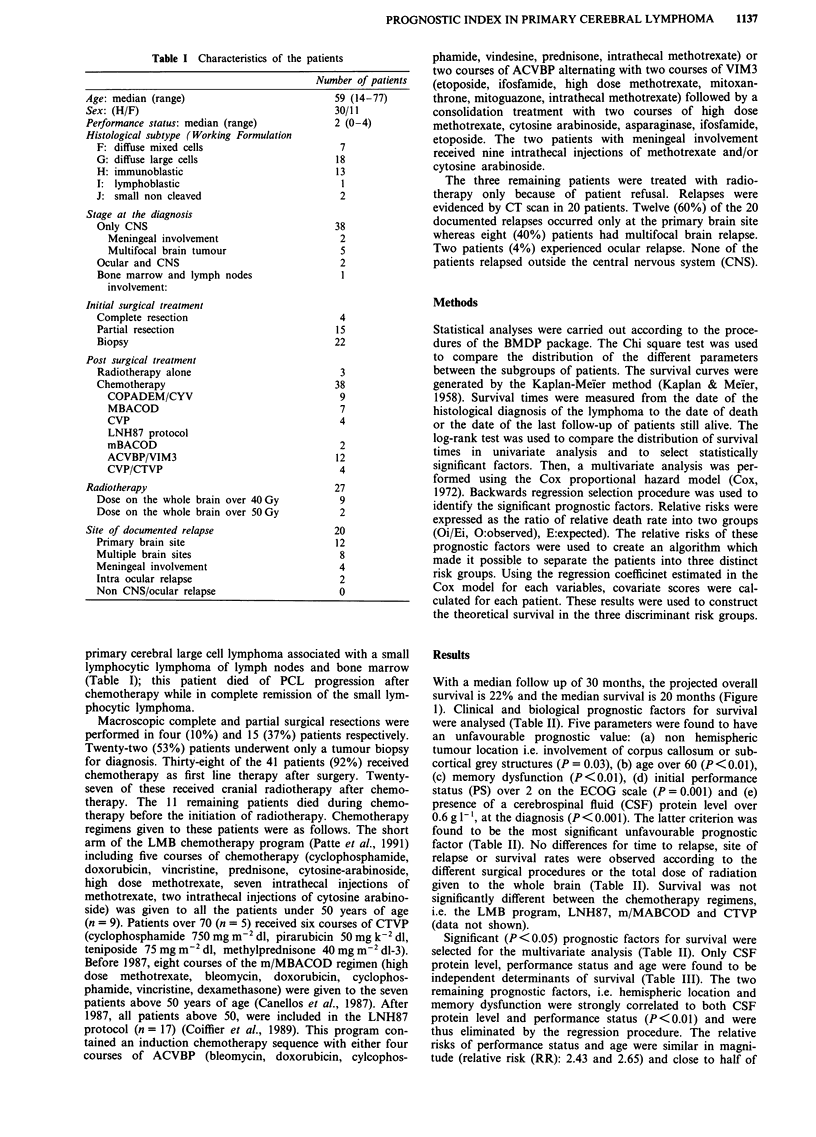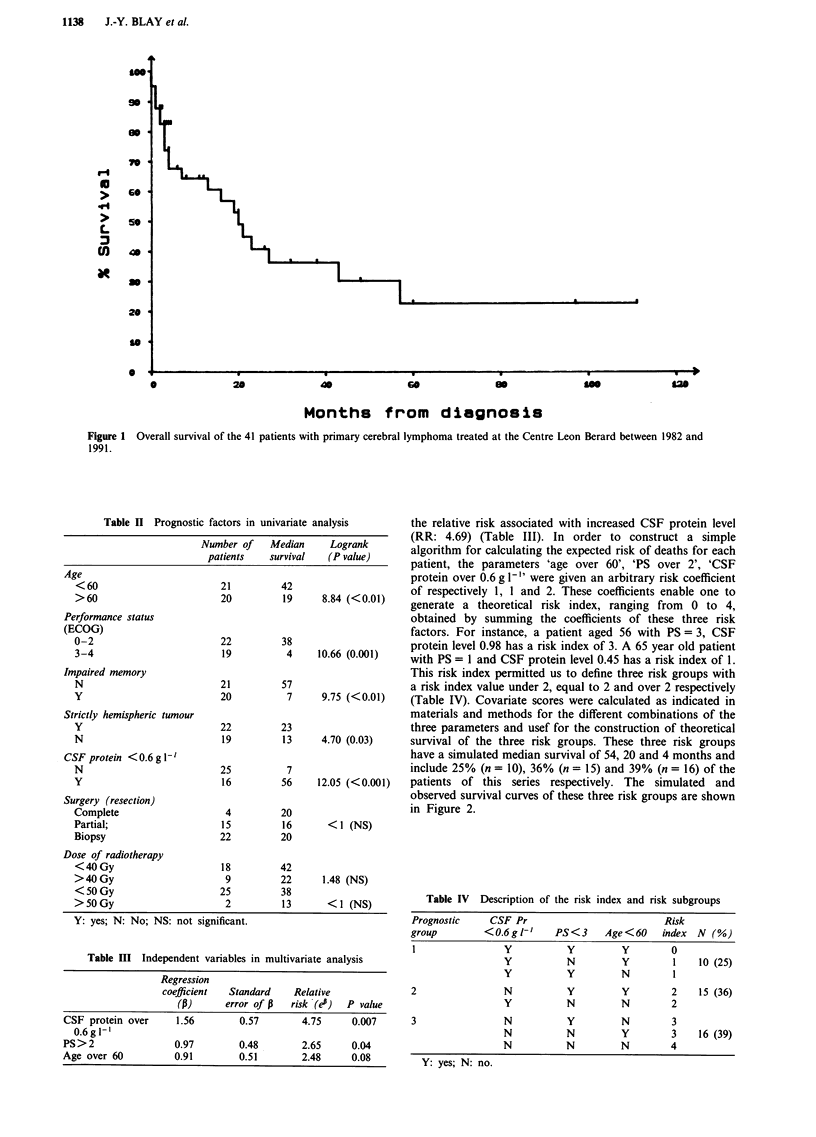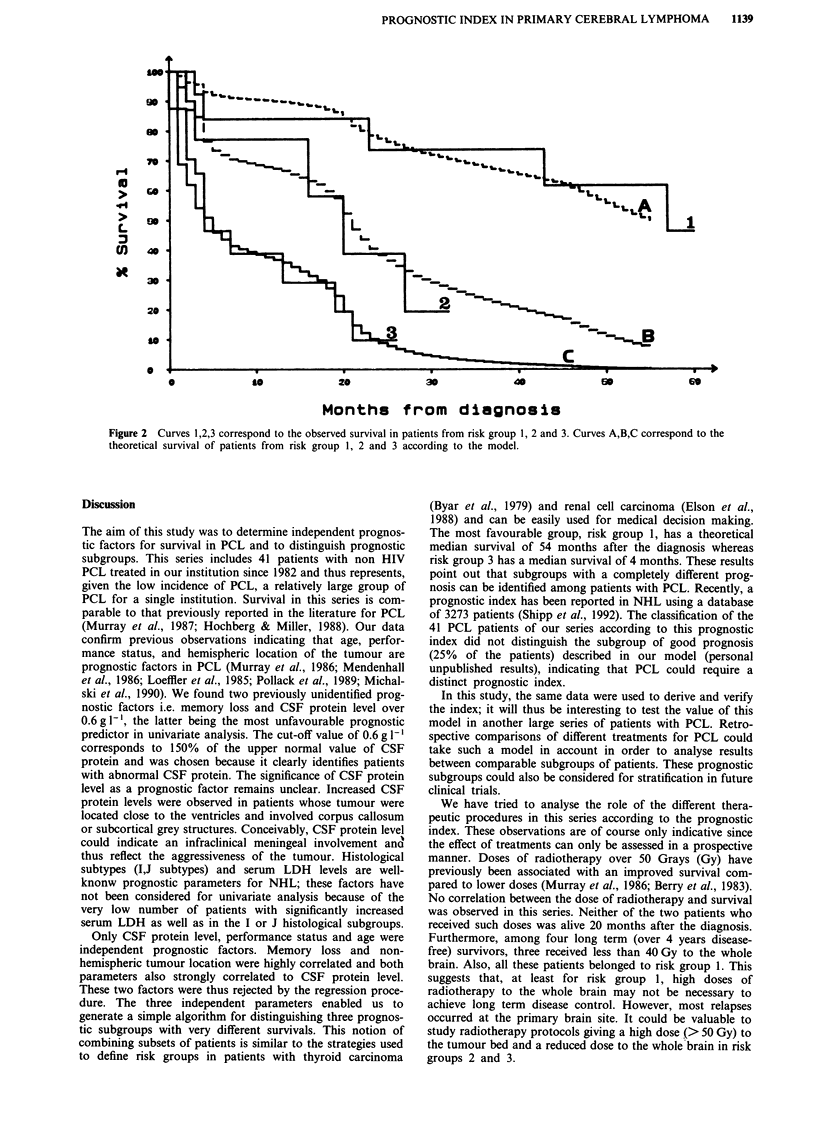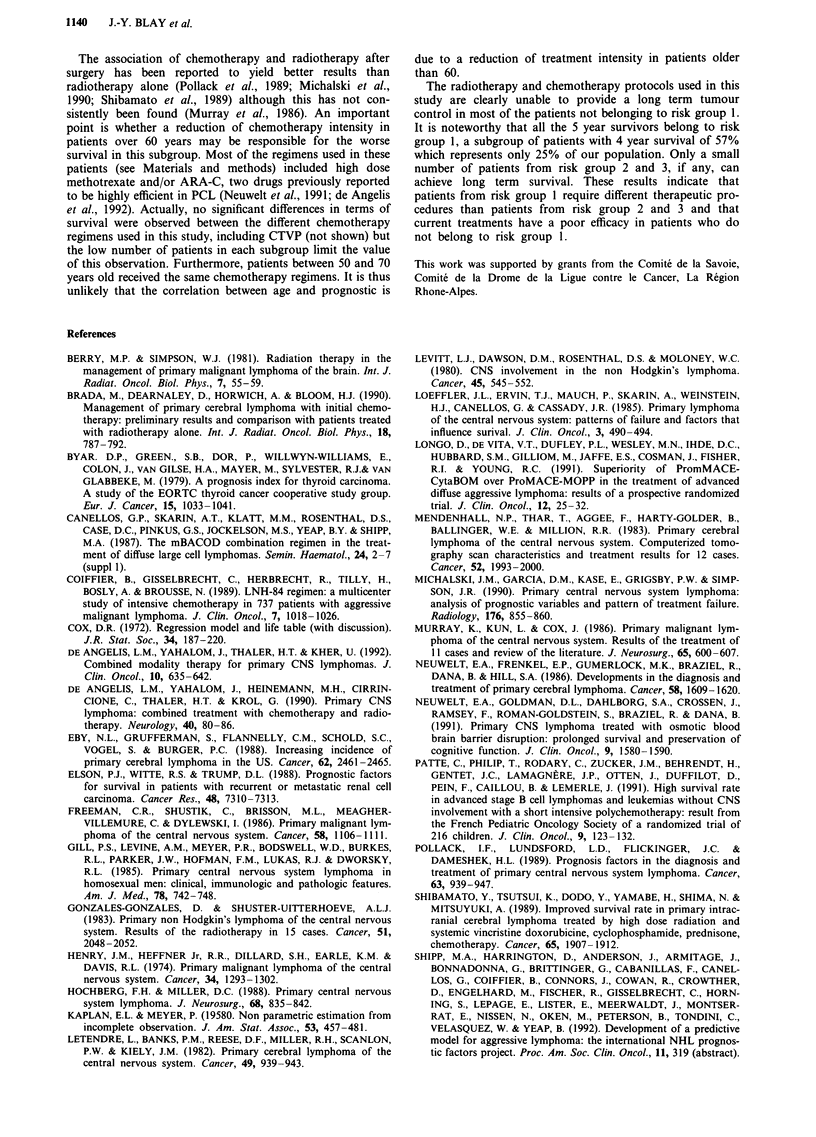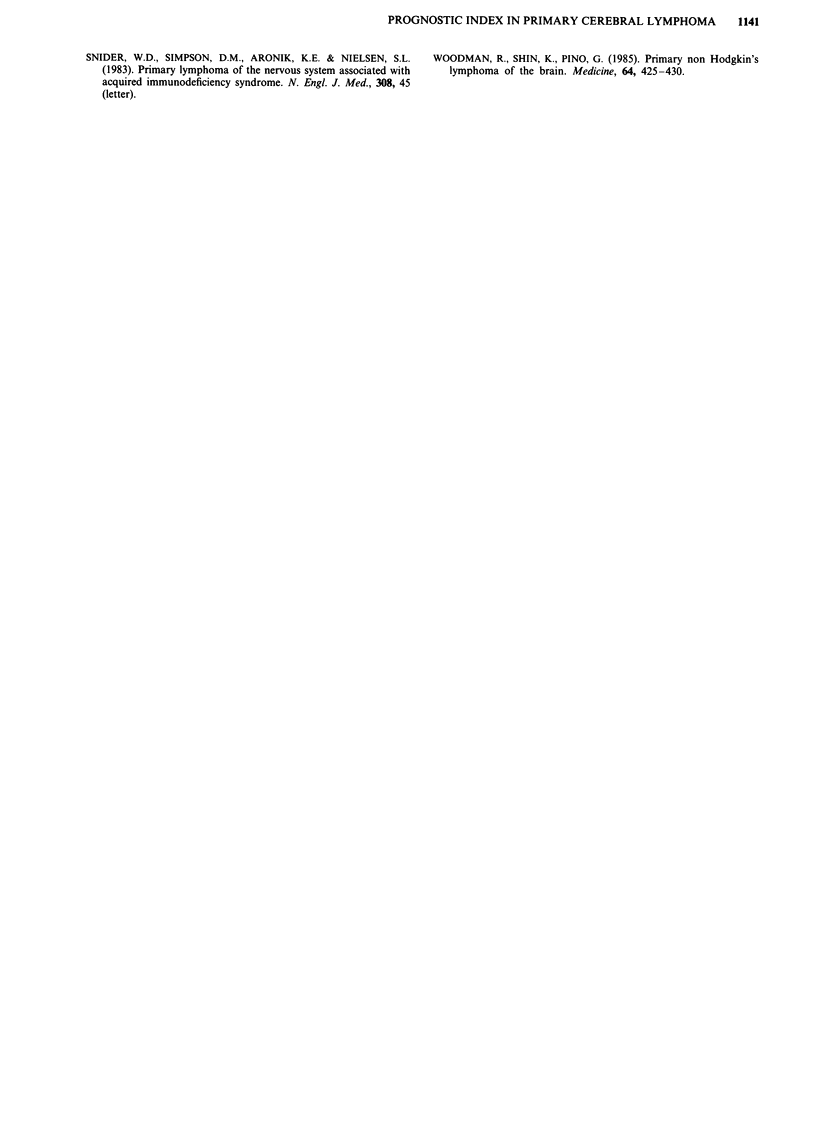# Multivariate analysis of prognostic factors in patients with non HIV-related primary cerebral lymphoma. A proposal for a prognostic scoring.

**DOI:** 10.1038/bjc.1993.209

**Published:** 1993-05

**Authors:** J. Y. Blay, C. Lasset, C. Carrie, F. Chauvin, B. Coiffier, C. Gisselbrecht, M. Clavel, P. Rebattu, M. Brunat-Mentigny, T. Philip

**Affiliations:** Department de Chimiothérapie massive et de greffe de moëlle, Centre Léon, Berard, France.

## Abstract

Between 1982 and 1991, 41 patients were treated for non HIV related primary cerebral lymphoma (PCL) in our institute. The purpose of this study was to perform a multivariate analysis of prognostic factors for survival in these patients. The presence of a CSF protein level over 0.6 g l-1 at diagnosis was found to be the most significant unfavourable prognostic factor in univariate analysis and had not previously been reported. Among the five significant prognostic factors at diagnosis, (age over 60 years, performance status--ECOG scale--over 2, memory dysfunction, non hemispheric tumour site, CSF protein level over 0.6 g l-1 at the diagnosis), three independent factors were identified in multivariate analysis: (1) CSF protein level (P = 0.007; RR = 4.7); (2) PS > 2 (P = 0.04, RR = 2.65); (3) age over 60 (P = 0.08; RR = 2.43). Using the regression coefficient of these three parameters, we determined a prognostic index which allowed us to distinguish three risk groups whose theoretical median survival is 4, 20 and 54, months respectively in patients with non HIV related PCL. These results indicate that PCL is an heterogeneous disease in terms of the prognostic in which three subgroups with discriminant survival can be identified.


 © Macmillan Press Ltd., 1993

### Multivariate analysis of prognostic factors in patients with non HIV-related primary cerebral lymphoma. A proposal for a prognostic scoring

J.-Y. Blay[1], C. Lasset[1], C. Carrie[1], F. Chauvin[1], B. Coiffier[2], C. Gisselbrecht[3], M. Clavel[1], P. Rebattu[1], M. Brunat-Mentigny[1], T. Philip[1] & P. Biron[1]

[1]*Department de chimiothérapie massive et de greffe de moëlle, département de radiothérapie, département de médicine. Centre Léon Berard, 28, Rue Laennec, 69008 Lyon;* [2]*Service d'hématologie, Centre Hospitalier Lyon Sud, 69310 Pierre-Benite;* [3]*Service d'hématologie, Hopital St Louis, 75010 Paris, France.*

**Summary** Between 1982 and 1991, 41 patients were treated for non HIV related primary cerebral lymphoma (PCL) in our institute. The purpose of this study was to perform a multivariate analysis of prognostic factors for survival in these patients. The presence of a CSF protein level over $0.6\ g\ l^{-1}$ at diagnosis was found to be the most significant unfavourable prognostic factor in univariate analysis and had not previously been reported. Among the five significant prognostic factors at diagnosis, (age over 60 years, performance status – ECOG scale – over 2, memory dysfunction, non hemispheric tumour site, CSF protein level over $0.6\ g\ l^{-1}$ at the diagnosis), three independent factors were identified in multivariate analysis: (1) CSF protein level ($P = 0.007$; RR = 4.7); (2) PS>2 ($P = 0.04$, RR = 2.65); (3) age over 60 ($P = 0.08$; RR = 2.43). Using the regression coefficient of these three parameters, we determined a prognostic index which allowed us to distinguish three risk groups whose theoretical median survival is 4, 20 and 54, months respectively in patients with non HIV related PCL. These results indicate that PCL is an heterogenous disease in terms of the prognostic in which three subgroups with discriminant survival can be identified.

Primary cerebral lymphoma (PCL) is a rare neoplasm accounting for approximately 0.7% of all lymphoma and less than 2% of all brain tumours (Henry *et al.*, 1974; Levitt *et al.*, 1980; Woodman *et al.*, 1985; Murray *et al.*, 1987; Hochberg & Miller, 1988). The incidence of PCL is significantly increased in patients with severe immunodeficiency including acquired immunodeficiency syndrome (AIDS) (Snyder *et al.*, 1983; Gill *et al.*, 1985). Recent reports indicate that the incidence of PCL is also increasing in apparently non-immunodeficient subjects, although the reason for these observations remains obscure (Eby *et al.*, 1988). In contrast to other non Hodgkin's lymphomas (Canellos *et al.*, 1987; Coiffer *et al.*, 1989; Longo *et al.*, 1991; Patte *et al.*, 1991), PCL has a poor prognosis in most of the series with treatment combining surgery with chemotherapy and/or radiotherapy (Murray *et al.*, 1987; Hochberg & Miller, 1988; Berry *et al.*, 1981; Letendre *et al.*, 1982; Gonzales-Gonzales *et al.*, 1983; Freeman *et al.*, 1986). However, intensive chemotherapy regimens including drugs that pass the blood-brain barrier appear to give improved results in recently published trials (De Angelis *et al.*, 1990; Neuwelt *et al.*, 1991; De Angelis *et al.*, 1992). Neuwelt *et al.* (1991) have described a regimen containing high dose methotrexate (HDMTX) and two alkylating agents given wtih osmotic blood-brain barrier disruption which yields a projected 40% survival and at 3 years. De Angelis *et al.* (1992) have reported a regimen combining HDMTX, cytosine arabinoside and cranial irradiation which yields 35% survival at 4 years.

Since PCL is a rare neoplasm, most of the series include a limited number of patients and the optimal treatment remains to be defined. Furthermore, comparison of therapeutic results between studies is complicated by the differences in the clinical presentations of this disease and the lack of knowledge of prognostic factors. Several univariate analyses, most involving less than 30 patients, have identified prognostic factors for survival in patients with PCL )Loeffler *et al.*, 1985; Murray *et al.*, 1986; Mendenhall *et al.*, 1986; Pollack *et al.*, 1989; Michalski *et al.*, 1990). Some of these prognostic factors are identical to those previously shown for aggressive lymphomas not involving the CNS, such as age, performance status at diagnosis, size of the lesion, and serum LDH levels.

Correspondence: J.-Y. Blay.
Received 29 September 1992; and in revised form 22 December 1992.

Specific prognostic factors for PCL, such as confinement of the tumour to cerebral hemispheres and extension of the tumour outside the brain have also been reported (Murray *et al.*, 1986; Loeffler *et al.*, 1985; Pollack *et al.*, 1989; Michalski *et al.*, 1990). Among therapeutic features, the dose of radiation (Murray *et al.*, 1986; Berry & Simpson, 1981; Pollack *et al.*, 1989; Brada *et al.*, 1990) and treatment with chemotherapy (Pollack *et al.*, 1989; Michalski *et al.*, 1990; Shibamato *et al.*, 1989) have been shown to correlate with survival. To date, however, no multivariate analysis of prognostic factors for survival in PCL has been reported.

Here, we report a retrospective analysis of 41 non-HIV non immunodeficient patients with PCL treated at the Centre Léon Bérard in Lyon between 1982 and 1991. Three independent,prognostic criteria were identified using the Cox model. We have determined an algorithm of relative risk which allows the identification of three different risk groups with a discriminant projected survival.

### Patients and methods

### *Patients*

Between 1982 and 1991, 46 patients with a non-HIV related PCL were recorded at the Centre Leon Berard (CLB). None of these patients had a known cause of immunodeficiency. Five of these 46 patients were referred only for radiotherapy or follow-up after an initial treatment performed outside of our institute and were not included in this analysis because of its insufficient data concerning their initial clinical status. Thus, 41 patients with PCL were diagnosed and treated at the CLB between 1982 and 1991. In 37 patients, HIV serology was performed and found negative. Four patients were not tested for HIV but none of them belonged to the previously reported risk groups and none had lymphopenia or experienced opportunistic infections. The clinical characteristics of these 41 patients are presented in Table I. All lymphomas were intermediate or high grade lymphomas with a majority of diffuse large cell lymphoma (47%). In most of the patients (85%), the tumour was confined to the brain. Four patients (10%) also had an ocular involvement diagnosed before ($n = 1$), synchronously ($n = 2$) or after ($n = 1$) the brain tumour as previously reported (Murray *et al.*, 1988; Hochberg & Simpson, 1988). One of the patients (5%) had a

Table I Characteristics of the patients

| | *Number of patients* |
|---|---|
| *Age*: median (range) | 59 (14–77) |
| *Sex*: (H/F) | 30/11 |
| *Performance status*: median (range) | 2 (0–4) |
| *Histological subtype (Working Formulation* | |
| F: diffuse mixed cells | 7 |
| G: diffuse large cells | 18 |
| H: immunoblastic | 13 |
| I: lymphoblastic | 1 |
| J: small non cleaved | 2 |
| *Stage at the diagnosis* | |
| Only CNS | 38 |
| Meningeal involvement | 2 |
| Multifocal brain tumour | 5 |
| Ocular and CNS | 2 |
| Bone marrow and lymph nodes involvement: | 1 |
| *Initial surgical treatment* | |
| Complete resection | 4 |
| Partial resection | 15 |
| Biopsy | 22 |
| *Post surgical treatment* | |
| Radiotherapy alone | 3 |
| Chemotherapy | 38 |
| COPADEM/CYV | 9 |
| MBACOD | 7 |
| CVP | 4 |
| LNH87 protocol | |
| mBACOD | 2 |
| ACVBP/VIM3 | 12 |
| CVP/CTVP | 4 |
| *Radiotherapy* | 27 |
| Dose on the whole brain over 40 Gy | 9 |
| Dose on the whole brain over 50 Gy | 2 |
| *Site of documented relapse* | 20 |
| Primary brain site | 12 |
| Multiple brain sites | 8 |
| Meningeal involvement | 4 |
| Intra ocular relapse | 2 |
| Non CNS/ocular relapse | 0 |

primary cerebral large cell lymphoma associated with a small lymphocytic lymphoma of lymph nodes and bone marrow (Table I); this patient died of PCL progression after chemotherapy while in complete remission of the small lymphocytic lymphoma.

Macroscopic complete and partial surgical resections were performed in four (10%) and 15 (37%) patients respectively. Twenty-two (53%) patients underwent only a tumour biopsy for diagnosis. Thirty-eight of the 41 patients (92%) received chemotherapy as first line therapy after surgery. Twenty-seven of these received cranial radiotherapy after chemotherapy. The 11 remaining patients died during chemotherapy before the initiation of radiotherapy. Chemotherapy regimens given to these patients were as follows. The short arm of the LMB chemotherapy program (Patte *et al.*, 1991) including five courses of chemotherapy (cyclophosphamide, doxorubicin, vincristine, prednisone, cytosine-arabinoside, high dose methotrexate, seven intrathecal injections of methotrexate, two intrathecal injections of cytosine arabinoside) was given to all the patients under 50 years of age ($n = 9$). Patients over 70 ($n = 5$) received six courses of CTVP (cyclophosphamide 750 mg m$^{-2}$ dl, pirarubicin 50 mg k$^{-2}$ dl, teniposide 75 mg m$^{-2}$ dl, methylprednisone 40 mg m$^{-2}$ dl-3). Before 1987, eight courses of the m/MBACOD regimen (high dose methotrexate, bleomycin, doxorubicin, cyclophosphamide, vincristine, dexamethasone) were given to the seven patients above 50 years of age (Canellos *et al.*, 1987). After 1987, all patients above 50, were included in the LNH87 protocol ($n = 17$) (Coiffier *et al.*, 1989). This program contained an induction chemotherapy sequence with either four courses of ACVBP (bleomycin, doxorubicin, cylcophosphamide, vindesine, prednisone, intrathecal methotrexate) or two courses of ACVBP alternating with two courses of VIM3 (etoposide, ifosfamide, high dose methotrexate, mitoxanthrone, mitoguazone, intrathecal methotrexate) followed by a consolidation treatment with two courses of high dose methotrexate, cytosine arabinoside, asparaginase, ifosfamide, etoposide. The two patients with meningeal involvement received nine intrathecal injections of methotrexate and/or cytosine arabinoside.

The three remaining patients were treated with radiotherapy only because of patient refusal. Relapses were evidenced by CT scan in 20 patients. Twelve (60%) of the 20 documented relapses occurred only at the primary brain site whereas eight (40%) patients had multifocal brain relapse. Two patients (4%) experienced ocular relapse. None of the patients relapsed outside the central nervous system (CNS).

### Methods

Statistical analyses were carried out according to the procedures of the BMDP package. The Chi square test was used to compare the distribution of the different parameters between the subgroups of patients. The survival curves were generated by the Kaplan-Meïer method (Kaplan & Meïer, 1958). Survival times were measured from the date of the histological diagnosis of the lymphoma to the date of death or the date of the last follow-up of patients still alive. The log-rank test was used to compare the distribution of survival times in univariate analysis and to select statistically significant factors. Then, a multivariate analysis was performed using the Cox proportional hazard model (Cox, 1972). Backwards regression selection procedure was used to identify the significant prognostic factors. Relative risks were expressed as the ratio of relative death rate into two groups (Oi/Ei, O:observed), E:expected). The relative risks of these prognostic factors were used to create an algorithm which made it possible to separate the patients into three distinct risk groups. Using the regression coefficinet estimated in the Cox model for each variables, covariate scores were calculated for each patient. These results were used to construct the theoretical survival in the three discriminant risk groups.

### Results

With a median follow up of 30 months, the projected overall survival is 22% and the median survival is 20 months (Figure 1). Clinical and biological prognostic factors for survival were analysed (Table II). Five parameters were found to have an unfavourable prognostic value: (a) non hemispheric tumour location i.e. involvement of corpus callosum or subcortical grey structures ($P = 0.03$), (b) age over 60 ($P < 0.01$), (c) memory dysfunction ($P < 0.01$), (d) initial performance status (PS) over 2 on the ECOG scale ($P = 0.001$) and (e) presence of a cerebrospinal fluid (CSF) protein level over 0.6 g l$^{-1}$, at the diagnosis ($P < 0.001$). The latter criterion was found to be the most significant unfavourable prognostic factor (Table II). No differences for time to relapse, site of relapse or survival rates were observed according to the different surgical procedures or the total dose of radiation given to the whole brain (Table II). Survival was not significantly different between the chemotherapy regimens, i.e. the LMB program, LNH87, m/MABCOD and CTVP (data not shown).

Significant ($P < 0.05$) prognostic factors for survival were selected for the multivariate analysis (Table II). Only CSF protein level, performance status and age were found to be independent determinants of survival (Table III). The two remaining prognostic factors, i.e. hemispheric location and memory dysfunction were strongly correlated to both CSF protein level and performance status ($P < 0.01$) and were thus eliminated by the regression procedure. The relative risks of performance status and age were similar in magnitude (relative risk (RR): 2.43 and 2.65) and close to half of

**Figure 1** Overall survival of the 41 patients with primary cerebral lymphoma treated at the Centre Leon Berard between 1982 and 1991.

**Table II** Prognostic factors in univariate analysis

| | *Number of patients* | *Median survival* | *Logrank (P value)* |
|---|---|---|---|
| *Age* | | | |
| <60 | 21 | 42 | |
| >60 | 20 | 19 | 8.84 (<0.01) |
| *Performance status* (ECOG) | | | |
| 0–2 | 22 | 38 | |
| 3–4 | 19 | 4 | 10.66 (0.001) |
| *Impaired memory* | | | |
| N | 21 | 57 | |
| Y | 20 | 7 | 9.75 (<0.01) |
| *Strictly hemispheric tumour* | | | |
| Y | 22 | 23 | |
| N | 19 | 13 | 4.70 (0.03) |
| *CSF protein* $<0.6\ g\ l^{-1}$ | | | |
| N | 25 | 7 | |
| Y | 16 | 56 | 12.05 (<0.001) |
| *Surgery (resection)* | | | |
| Complete | 4 | 20 | |
| Partial; | 15 | 16 | <1 (NS) |
| Biopsy | 22 | 20 | |
| *Dose of radiotherapy* | | | |
| <40 Gy | 18 | 42 | |
| >40 Gy | 9 | 22 | 1.48 (NS) |
| <50 Gy | 25 | 38 | |
| >50 Gy | 2 | 13 | <1 (NS) |

Y: yes; N: No; NS: not significant.

**Table III** Independent variables in multivariate analysis

| | *Regression coefficient (β)* | *Standard error of β* | *Relative risk ($e^{\beta}$)* | *P value* |
|---|---|---|---|---|
| CSF protein over $0.6\ g\ l^{-1}$ | 1.56 | 0.57 | 4.75 | 0.007 |
| PS>2 | 0.97 | 0.48 | 2.65 | 0.04 |
| Age over 60 | 0.91 | 0.51 | 2.48 | 0.08 |

the relative risk associated with increased CSF protein level (RR: 4.69) (Table III). In order to construct a simple algorithm for calculating the expected risk of deaths for each patient, the parameters 'age over 60', 'PS over 2', 'CSF protein over $0.6\ g\ l^{-1}$' were given an arbitrary risk coefficient of respectively 1, 1 and 2. These coefficients enable one to generate a theoretical risk index, ranging from 0 to 4, obtained by summing the coefficients of these three risk factors. For instance, a patient aged 56 with PS = 3, CSF protein level 0.98 has a risk index of 3. A 65 year old patient with PS = 1 and CSF protein level 0.45 has a risk index of 1. This risk index permitted us to define three risk groups with a risk index value under 2, equal to 2 and over 2 respectively (Table IV). Covariate scores were calculated as indicated in materials and methods for the different combinations of the three parameters and usef for the construction of theoretical survival of the three risk groups. These three risk groups have a simulated median survival of 54, 20 and 4 months and include 25% ($n$ = 10), 36% ($n$ = 15) and 39% ($n$ = 16) of the patients of this series respectively. The simulated and observed survival curves of these three risk groups are shown in Figure 2.

**Table IV** Description of the risk index and risk subgroups

| *Prognostic group* | *CSF Pr* $<0.6\ g\ l^{-1}$ | *PS<3* | *Age<60* | *Risk index* | *N (%)* |
|---|---|---|---|---|---|
| 1 | Y | Y | Y | 0 | |
| | Y | N | Y | 1 | 10 (25) |
| | Y | Y | N | 1 | |
| 2 | N | Y | Y | 2 | 15 (36) |
| | Y | N | N | 2 | |
| 3 | N | Y | N | 3 | |
| | N | N | Y | 3 | 16 (39) |
| | N | N | N | 4 | |

Y: yes; N: no.

**Figure 2** Curves 1,2,3 correspond to the observed survival in patients from risk group 1, 2 and 3. Curves A,B,C correspond to the theoretical survival of patients from risk group 1, 2 and 3 according to the model.

### Discussion

The aim of this study was to determine independent prognostic factors for survival in PCL and to distinguish prognostic subgroups. This series includes 41 patients with non HIV PCL treated in our institution since 1982 and thus represents, given the low incidence of PCL, a relatively large group of PCL for a single institution. Survival in this series is comparable to that previously reported in the literature for PCL (Murray *et al.*, 1987; Hochberg & Miller, 1988). Our data confirm previous observations indicating that age, performance status, and hemispheric location of the tumour are prognostic factors in PCL (Murray *et al.*, 1986; Mendenhall *et al.*, 1986; Loeffler *et al.*, 1985; Pollack *et al.*, 1989; Michalski *et al.*, 1990). We found two previously unidentified prognostic factors i.e. memory loss and CSF protein level over $0.6\,g\,l^{-1}$, the latter being the most unfavourable prognostic predictor in univariate analysis. The cut-off value of $0.6\,g\,l^{-1}$ corresponds to 150% of the upper normal value of CSF protein and was chosen because it clearly identifies patients with abnormal CSF protein. The significance of CSF protein level as a prognostic factor remains unclear. Increased CSF protein levels were observed in patients whose tumour were located close to the ventricles and involved corpus callosum or subcortical grey structures. Conceivably, CSF protein level could indicate an infraclinical meningeal involvement and thus reflect the aggressiveness of the tumour. Histological subtypes (I,J subtypes) and serum LDH levels are well-knonw prognostic parameters for NHL; these factors have not been considered for univariate analysis because of the very low number of patients with significantly increased serum LDH as well as in the I or J histological subgroups.

Only CSF protein level, performance status and age were independent prognostic factors. Memory loss and non-hemispheric tumour location were highly correlated and both parameters also strongly correlated to CSF protein level. These two factors were thus rejected by the regression procedure. The three independent parameters enabled us to generate a simple algorithm for distinguishing three prognostic subgroups with very different survivals. This notion of combining subsets of patients is similar to the strategies used to define risk groups in patients with thyroid carcinoma (Byar *et al.*, 1979) and renal cell carcinoma (Elson *et al.*, 1988) and can be easily used for medical decision making. The most favourable group, risk group 1, has a theoretical median survival of 54 months after the diagnosis whereas risk group 3 has a median survival of 4 months. These results point out that subgroups with a completely different prognosis can be identified among patients with PCL. Recently, a prognostic index has been reported in NHL using a database of 3273 patients (Shipp *et al.*, 1992). The classification of the 41 PCL patients of our series according to this prognostic index did not distinguish the subgroup of good prognosis (25% of the patients) described in our model (personal unpublished results), indicating that PCL could require a distinct prognostic index.

In this study, the same data were used to derive and verify the index; it will thus be interesting to test the value of this model in another large series of patients with PCL. Retrospective comparisons of different treatments for PCL could take such a model in account in order to analyse results between comparable subgroups of patients. These prognostic subgroups could also be considered for stratification in future clinical trials.

We have tried to analyse the role of the different therapeutic procedures in this series according to the prognostic index. These observations are of course only indicative since the effect of treatments can only be assessed in a prospective manner. Doses of radiotherapy over 50 Grays (Gy) have previously been associated with an improved survival compared to lower doses (Murray *et al.*, 1986; Berry *et al.*, 1983). No correlation between the dose of radiotherapy and survival was observed in this series. Neither of the two patients who received such doses was alive 20 months after the diagnosis. Furthermore, among four long term (over 4 years disease-free) survivors, three received less than 40 Gy to the whole brain. Also, all these patients belonged to risk group 1. This suggests that, at least for risk group 1, high doses of radiotherapy to the whole brain may not be necessary to achieve long term disease control. However, most relapses occurred at the primary brain site. It could be valuable to study radiotherapy protocols giving a high dose ($>50$ Gy) to the tumour bed and a reduced dose to the whole brain in risk groups 2 and 3.

The association of chemotherapy and radiotherapy after surgery has been reported to yield better results than radiotherapy alone (Pollack *et al.*, 1989; Michalski *et al.*, 1990; Shibamato *et al.*, 1989) although this has not consistently been found (Murray *et al.*, 1986). An important point is whether a reduction of chemotherapy intensity in patients over 60 years may be responsible for the worse survival in this subgroup. Most of the regimens used in these patients (see Materials and methods) included high dose methotrexate and/or ARA-C, two drugs previously reported to be highly efficient in PCL (Neuwelt *et al.*, 1991; de Angelis *et al.*, 1992). Actually, no significant differences in terms of survival were observed between the different chemotherapy regimens used in this study, including CTVP (not shown) but the low number of patients in each subgroup limit the value of this observation. Furthermore, patients between 50 and 70 years old received the same chemotherapy regimens. It is thus unlikely that the correlation between age and prognostic is due to a reduction of treatment intensity in patients older than 60.

The radiotherapy and chemotherapy protocols used in this study are clearly unable to provide a long term tumour control in most of the patients not belonging to risk group 1. It is noteworthy that all the 5 year survivors belong to risk group 1, a subgroup of patients with 4 year survival of 57% which represents only 25% of our population. Only a small number of patients from risk group 2 and 3, if any, can achieve long term survival. These results indicate that patients from risk group 1 require different therapeutic procedures than patients from risk group 2 and 3 and that current treatments have a poor efficacy in patients who do not belong to risk group 1.

This work was supported by grants from the Comité de la Savoie, Comité de la Drome de la Ligue contre le Cancer, La Région Rhone-Alpes.

### References

BERRY, M.P. & SIMPSON, W.J. (1981). Radiation therapy in the management of primary malignant lymphoma of the brain. *Int. J. Radiat. Oncol. Biol. Phys.*, **7**, 55–59.

BRADA, M., DEARNALEY, D., HORWICH, A. & BLOOM, H.J. (1990). Management of primary cerebral lymphoma with initial chemotherapy: preliminary results and comparison with patients treated with radiotherapy alone. *Int. J. Radiat. Oncol. Biol. Phys.*, **18**, 787–792.

BYAR. D.P., GREEN., S.B., DOR, P., WILLWYN-WILLIAMS, E., COLON, J., VAN GILSE, H.A., MAYER, M., SYLVESTER, R.J.& VAN GLABBEKE, M. (1979). A prognosis index for thyroid carcinoma. A study of the EORTC thyroid cancer cooperative study group. *Eur. J. Cancer*, **15**, 1033–1041.

CANELLOS, G.P., SKARIN, A.T., KLATT, M.M., ROSENTHAL, D.S., CASE, D.C., PINKUS, G.S., JOCKELSON, M.S., YEAP, B.Y. & SHIPP, M.A. (1987). The mBACOD combination regimen in the treatment of diffuse large cell lymphomas. *Semin. Haematol.*, **24**, 2–7 (suppl 1).

COIFFIER, B., GISSELBRECHT, C., HERBRECHT, R., TILLY, H., BOSLY, A. & BROUSSE, N. (1989). LNH-84 regimen: a multicenter study of intensive chemotherapy in 737 patients with aggressive malignant lymphoma. *J. Clin. Oncol.*, **7**, 1018–1026.

COX, D.R. (1972). Regression model and life table (with discussion). *J.R. Stat. Soc.*, **34**, 187–220.

DE ANGELIS, L.M., YAHALOM, J., THALER, H.T. & KHER, U. (1992). Combined modality therapy for primary CNS lymphomas. *J. Clin. Oncol.*, **10**, 635–642.

DE ANGELIS, L.M., YAHALOM, J., HEINEMANN, M.H., CIRRINCIONE, C., THALER, H.T. & KROL, G. (1990). Primary CNS lymphoma: combined treatment with chemotherapy and radiotherapy. *Neurology*, **40**, 80–86.

EBY, N.L., GRUFFERMAN, S., FLANNELLY, C.M., SCHOLD, S.C., VOGEL, S. & BURGER, P.C. (1988). Increasing incidence of primary cerebral lymphoma in the US. *Cancer*, **62**, 2461–2465.

ELSON, P.J., WITTE, R.S. & TRUMP, D.L. (1988). Prognostic factors for survival in patients with recurrent or metastatic renal cell carcinoma. *Cancer Res.*, **48**, 7310–7313.

FREEMAN, C.R., SHUSTIK, C., BRISSON, M.L., MEAGHER-VILLEMURE, C. & DYLEWSKI, I. (1986). Primary malignant lymphoma of the central nervous system. *Cancer*, **58**, 1106–1111.

GILL, P.S., LEVINE, A.M., MEYER, P.R., BODSWELL, W.D., BURKES, R.L., PARKER, J.W., HOFMAN, F.M., LUKAS, R.J. & DWORSKY, R.L. (1985). Primary central nervous system lymphoma in homosexual men: clinical, immunologic and pathologic features. *Am. J. Med.*, **78**, 742–748.

GONZALES-GONZALES, D. & SHUSTER-UITTERHOEVE, A.L.J. (1983). Primary non Hodgkin's lymphoma of the central nervous system. Results of the radiotherapy in 15 cases. *Cancer*, **51**, 2048–2052.

HENRY, J.M., HEFFNER Jr, R.R., DILLARD, S.H., EARLE, K.M. & DAVIS, R.L. (1974). Primary malignant lymphoma of the central nervous system. *Cancer*, **34**, 1293–1302.

HOCHBERG, F.H. & MILLER, D.C. (1988). Primary central nervous system lymphoma. *J. Neurosurg.*, **68**, 835–842.

KAPLAN, E.L. & MEYER, P. (19580. Non parametric estimation from incomplete observation. *J. Am. Stat. Assoc.*, **53**, 457–481.

LETENDRE, L., BANKS, P.M., REESE, D.F., MILLER, R.H., SCANLON, P.W. & KIELY, J.M. (1982). Primary cerebral lymphoma of the central nervous system. *Cancer*, **49**, 939–943.

LEVITT, L.J., DAWSON, D.M., ROSENTHAL, D.S. & MOLONEY, W.C. (1980). CNS involvement in the non Hodgkin's lymphoma. *Cancer*, **45**, 545–552.

LOEFFLER, J.L., ERVIN, T.J., MAUCH, P., SKARIN, A., WEINSTEIN, H.J., CANELLOS, G. & CASSADY, J.R. (1985). Primary lymphoma of the central nervous system: patterns of failure and factors that influence surival. *J. Clin. Oncol.*, **3**, 490–494.

LONGO, D., DE VITA, V.T., DUFLEY, P.L., WESLEY, M.N., IHDE, D.C., HUBBARD, S.M., GILLIOM, M., JAFFE, E.S., COSMAN, J., FISHER, R.I. & YOUNG, R.C. (1991). Superiority of PromMACE-CytaBOM over ProMACE-MOPP in the treatment of advanced diffuse aggressive lymphoma: results of a prospective randomized trial. *J. Clin. Oncol.*, **12**, 25–32.

MENDENHALL, N.P., THAR, T., AGGEE, F., HARTY-GOLDER, B., BALLINGER, W.E. & MILLION, R.R. (1983). Primary cerebral lymphoma of the central nervous system. Computerized tomography scan characteristics and treatment results for 12 cases. *Cancer*, **52**, 1993–2000.

MICHALSKI, J.M., GARCIA, D.M., KASE, E., GRIGSBY, P.W. & SIMPSON, J.R. (1990). Primary central nervous system lymphoma: analysis of prognostic variables and pattern of treatment failure. *Radiology*, **176**, 855–860.

MURRAY, K., KUN, L. & COX, J. (1986). Primary malignant lymphoma of the central nervous system. Results of the treatment of 11 cases and review of the literature. *J. Neurosurg.*, **65**, 600–607.

NEUWELT, E.A., FRENKEL, E.P., GUMERLOCK, M.K., BRAZIEL, R., DANA, B. & HILL, S.A. (1986). Developments in the diagnosis and treatment of primary cerebral lymphoma. *Cancer*, **58**, 1609–1620.

NEUWELT, E.A., GOLDMAN, D.L., DAHLBORG, S.A., CROSSEN, J., RAMSEY, F., ROMAN-GOLDSTEIN, S., BRAZIEL, R. & DANA, B. (1991). Primary CNS lymphoma treated with osmotic blood brain barrier disruption: prolonged survival and preservation of cognitive function. *J. Clin. Oncol.*, **9**, 1580–1590.

PATTE, C., PHILIP, T., RODARY, C., ZUCKER, J.M., BEHRENDT, H., GENTET, J.C., LAMAGNÈRE, J.P., OTTEN, J., DUFFILOT, D., PEIN, F., CAILLOU, B. & LEMERLE, J. (1991). High survival rate in advanced stage B cell lymphomas and leukemias without CNS involvement with a short intensive polychemotherapy: result from the French Pediatric Oncology Society of a randomized trial of 216 children. *J. Clin. Oncol.*, **9**, 123–132.

POLLACK, I.F., LUNDSFORD, L.D., FLICKINGER, J.C. & DAMESHEK, H.L. (1989). Prognosis factors in the diagnosis and treatment of primary central nervous system lymphoma. *Cancer*, **63**, 939–947.

SHIBAMATO, Y., TSUTSUI, K., DODO, Y., YAMABE, H., SHIMA, N. & MITSUYUKI, A. (1989). Improved survival rate in primary intracranial cerebral lymphoma treated by high dose radiation and systemic vincristine doxorubicine, cyclophosphamide, prednisone, chemotherapy. *Cancer*, **65**, 1907–1912.

SHIPP, M.A., HARRINGTON, D., ANDERSON, J., ARMITAGE, J., BONNADONNA, G., BRITTINGER, G., CABANILLAS, F., CANELLOS, G., COIFFIER, B., CONNORS, J., COWAN, R., CROWTHER, D., ENGELHARD, M., FISCHER, R., GISSELBRECHT, C., HORNING, S., LEPAGE, E., LISTER, E., MEERWALDT, J., MONTSERRAT, E., NISSEN, N., OKEN, M., PETERSON, B., TONDINI, C., VELASQUEZ, W. & YEAP, B. (1992). Development of a predictive model for aggressive lymphoma: the international NHL prognostic factors project. *Proc. Am. Soc. Clin. Oncol.*, **11**, 319 (abstract).

SNIDER, W.D., SIMPSON, D.M., ARONIK, K.E. & NIELSEN, S.L. (1983). Primary lymphoma of the nervous system associated with acquired immunodeficiency syndrome. *N. Engl. J. Med.*, **308**, 45 (letter).

WOODMAN, R., SHIN, K., PINO, G. (1985). Primary non Hodgkin's lymphoma of the brain. *Medicine*, **64**, 425–430.